# Ribosomal Protein S6 Hypofunction in Postmortem Human Brain Links mTORC1-Dependent Signaling and Schizophrenia

**DOI:** 10.3389/fphar.2020.00344

**Published:** 2020-03-24

**Authors:** Inés Ibarra-Lecue, Rebeca Diez-Alarcia, Benito Morentin, J. Javier Meana, Luis F. Callado, Leyre Urigüen

**Affiliations:** ^1^Department of Pharmacology, University of the Basque Country UPV/EHU and Centro de Investigación Biomédica en Red de Salud Mental CIBERSAM, Leioa, Spain; ^2^Biocruces Bizkaia Health Research Institute, Barakaldo, Spain; ^3^Section of Forensic Pathology, Basque Institute of Legal Medicine, Bilbao, Spain

**Keywords:** ribosomal protein S6, schizophrenia, mTORC1, postmortem tissue, antipsychotics

## Abstract

The mechanistic target of rapamycin (also known as mammalian target of rapamycin) (mTOR)-dependent signaling pathway plays an important role in protein synthesis, cell growth, and proliferation, and has been linked to the development of the central nervous system. Recent studies suggest that mTOR signaling pathway dysfunction could be involved in the etiopathogenesis of schizophrenia. The main goal of this study was to evaluate the status of mTOR signaling pathway in postmortem prefrontal cortex (PFC) samples of subjects with schizophrenia. For this purpose, we quantified the protein expression and phosphorylation status of the mTOR downstream effector ribosomal protein S6 as well as other pathway interactors such as Akt and GSK3β. Furthermore, we quantified the status of these proteins in the brain cortex of rats chronically treated with the antipsychotics haloperidol, clozapine, or risperidone. We found a striking decrease in the expression of total S6 and in its active phosphorylated form phospho-S6 (Ser235/236) in the brain of subjects with schizophrenia compared to matched controls. The chronic treatment with the antipsychotics haloperidol and clozapine affected both the expression of GSK3β and the activation of Akt [phospho-Akt (Ser473)] in rat brain cortex, while no changes were observed in S6 and phospho-S6 (Ser235/236) protein expression with any antipsychotic treatment. These findings provide further evidence for the involvement of the mTOR-dependent signaling pathway in schizophrenia and suggest that a hypofunctional S6 may have a role in the etiopathogenesis of this disorder.

## Introduction

The mechanistic target of rapamycin (mTOR), also known as mammalian target of rapamycin, represents a critical integrator of neuronal activity and synaptic inputs that plays a role in schizophrenia and other neuropsychiatric diseases (Costa-Mattioli and Monteggia, 2013; Gururajan and Van Den Buuse, 2014; Pham et al., 2016; Chadha and Meador-Woodruff, 2020). The canonical activation of mTOR pathway results from an activation cascade of upstream proteins, including receptor tyrosine kinases, phosphatidylinositol-3-kinase (PI3K), and Akt (also known as protein kinase B, PKB). Akt is a serine/threonine kinase classically related to cellular survival, growth and proliferation, prevention of apoptosis, and cancer progression (Kennedy et al., 1997; Manning and Cantley, 2007). The Akt signaling is one of the key outcomes of the activation of PI3K. It supposes an intermediator of cellular pathways activated in response to different extracellular stimuli including growth factors, insulin, or Gβ/γ subunits of G-protein coupled receptors (GPCR) (Burgering and Coffer, 1995; Murga et al., 1998). In neurons, Akt regulates a wide variety of processes such as neural survival and architecture, axonal growth, or synaptic strength control.

A genetic association between variants of the AKT1 gene and schizophrenia has been shown in several populations (Ikeda et al., 2004; Schwab et al., 2005; Thiselton et al., 2008; Mathur et al., 2010). Moreover, AKT allelic variants have been also associated with cognitive impairments and morphological abnormalities in neural networks of the prefrontal cortex (PFC) (Pietilainen et al., 2009). Additionally, a deficiency in Akt protein function seems to promote alterations in PFC and schizophrenia-like behaviors in animal models (Lai et al., 2006). However, literature tackling Akt expression or functional/phosphorylation status in human brain shows discrepant results (Emamian et al., 2004; Zhao et al., 2006; Thiselton et al., 2008; Balu et al., 2012; Hino et al., 2016; McGuire et al., 2017). Akt negatively regulates the activity of GSK3β, which strongly influences the neural function, including gene expression, neuronal architecture, plasticity, and survival. In this context, a lower expression/density and activity of Akt (Zhao et al., 2006) and GSK3β (Emamian et al., 2004) has been described in the PFC of subjects with schizophrenia, although there are contradictory reports (Ide et al., 2006; Amar et al., 2008; Kitagishi et al., 2012; McGuire et al., 2017).

mTOR is a serine/threonine kinase that binds to several interacting proteins, forming two different heteromeric complexes, mTORC1 and mTORC2. The mTORC1 regulates protein synthesis by phosphorylating downstream effectors directly involved in translation control. Once active, mTORC1 phosphorylates ribosomal protein S6 kinase (S6K) and eukaryotic translation initiation factor 4E (eIF-4E)-binding protein 1 (4E-BP1), both involved in the translational machinery for protein synthesis (Meyuhas, 2015; Qin et al., 2016). The first identified substrate of S6K was ribosomal protein S6, a component of the 40S ribosome, which phosphorylation is considered as a readout of mTORC1 activity, and has become a widely used marker of neuronal activity. Phosphorylation of S6 regulates the translation of a subset of mRNA in the central nervous system (CNS) (Puighermanal et al., 2017) and regulates diverse biological processes important for cell growth, ribosome biogenesis, and protein translation (Ruvinsky and Meyuhas, 2006). This downstream effector of mTORC1 is regulated by several environmental factors and extracellular stimuli, and in the CNS it regulates neuronal plasticity associated to cognitive processes, such as learning and memory (Meyuhas, 2015; Pirbhoy et al., 2016; Pirbhoy et al., 2017).

PI3K/Akt/GSK3/mTOR pathway has been linked to the development of the CNS, neuronal growth, maintenance, and proliferation (Kitagishi et al., 2012). Moreover, the dysfunction of mTORC1/S6 pathway may contribute to an aberrant dendritic reorganization and the loss of dendritic spines, what finally would lead to dysfunctions in synaptic connectivity (Antion et al., 2008; Magdalon et al., 2017; Chadha and Meador-Woodruff, 2020). Furthermore, diverse molecules that have been previously implicated in schizophrenia, such as glutamate, reelin, BDNF, serotonin, and/or their respective receptors can lead to either over‐activation or inhibition of this signaling pathway (Gururajan and Van Den Buuse, 2014). All these data suggest that this pathway could have a significant role in schizophrenia (Costa-Mattioli and Monteggia, 2013; Gururajan and Van Den Buuse, 2014; Chadha and Meador-Woodruff, 2020).

To go through this hypothesis, we quantified the protein expression and phosphorylation of the mTOR downstream effector ribosomal protein S6 as well as other pathway interactors such as Akt and GSK3β, in postmortem PFC from subjects with schizophrenia and control subjects. To decipher the effect of the long-term treatment with different antipsychotics on this pathway, we also evaluated the status of the target proteins in the brain cortex of rats chronically treated with the antipsychotics haloperidol, clozapine or risperidone.

## Materials and Methods

### Postmortem Human Brain Samples

Human brain samples were obtained at autopsies performed in the Basque Institute of Legal Medicine, Bilbao, in compliance with policies of research and ethical boards for postmortem brain studies. Deaths were subjected to retrospective searching for previous medical diagnosis and treatment using examiner’s information and records from hospitals and mental health centers. Brain samples of 28 subjects with an antemortem diagnosis of schizophrenia according to the Diagnostic and Statistical Manual of Mental Disorders (DSM-IV-TR) (American Psychiatric Association, 2000) were matched to samples of 28 control subjects in a paired design. Control subjects were chosen based on the absence of diagnosis of neuropsychiatric disorders or drug abuse, and an appropriate gender, age, and postmortem interval (time between death and tissue dissection/freezing; PMI) to match each subject in the schizophrenia group. A blood toxicological screening was performed in all the subjects to determine the presence of antipsychotics, other drugs, and ethanol (National Institute of Toxicology, Madrid, Spain). Schizophrenia subjects (SZ) were divided into two groups according to the absence [antipsychotic-free (AP-F, n = 17)] or presence [antipsychotic-treated (AP-T, n = 11)] of antipsychotic drugs in blood at the time of death. Demographic characteristics and PMI values did not significantly differ between schizophrenia and control groups, nor between AP-F and AP-T subjects (**Table 1**). Samples of dorsolateral PFC (DLPFC) were dissected at autopsy (0.5**–**1 g tissue) following standard procedures (Rajkowska and Goldman-Rakic, 1995), and immediately stored at −80°C until assay. A demographic full description of subjects with schizophrenia (AP-F and AP-T) and their individually matched controls is summarized in **Supplementary Tables 1** and **2**.

**Table 1:** Demographic characteristics of postmortem human PFC samples.

Subjects with schizophrenia (n = 28) and matched controls (n = 28)				
Group	Gender (M/F)	Age (years)	PMI (h)	Storage time (months)
**Schizophrenia**	23M/5F	38.7 ± 2.1	17.8 ± 2.4	51.1 ± 7.7
AP-F (n = 17)	14M/3F	38.6 ± 2.4	19.2 ± 3.6	54.2 ± 11.4
AP-T (n = 11)	9M/2F	38.9 ± 3.9	16.1 ± 2.1	46.3 ± 9.1
**Control group**	22M/6F	38.8 ± 2.1	22.5 ± 2.1	45.8 ± 9.3

PFC, prefrontal cortex; M, male; F, female; PMI, postmortem interval; AP-F, antipsychotic-free; AP-T, antipsychotic-treated. Mean ± SEM.

### Animals and Treatments

Male Sprague-Dawley rats (222–318 g) (Animal Facility of the University of the Basque Country, Leioa, Spain) were housed on a 12-h light/dark cycle at 22**°**C and 60% humidity with food and water available *ad libitum*. All experimental procedures were performed in accordance with the European Union Directive 2010/63/EU and approved by the Ethic Committee for Animal Welfare of the University of the Basque Country, UPV/EHU (CEBA 188/2011). Animals were treated twice-a-day (i.p., volume 1 ml/kg), during 21 days, with saline (1 ml/kg), clozapine (5 mg/kg) (Tocris. Bristol, UK), risperidone (0.5 mg/kg), or haloperidol (0.5 mg/kg) (Sigma Aldrich^®^. Missouri, USA). These doses have been previously used in the literature (Parikh et al., 2004) and yield serum concentrations comparable to those observed in humans receiving these treatments (Kapur et al., 2003). Clozapine was dissolved in a few drops of glacial acetic acid (Panreac Química S.A. Barcelona, Spain) and prepared in distilled water; risperidone and haloperidol were dissolved in saline. After 48 h (clozapine and risperidone) or 72 h (haloperidol and saline) washout-period, rats were sacrificed, brains removed, and cortex dissected and stored at −80°C until assay.

### Preparation of Total Homogenates

Brain homogenates were obtained as previously described (Urigüen et al., 2009) with minor modifications. Briefly, human PFC (~200 mg) or rat cortex (~130 mg) tissue samples were thawed at 4°C and homogenized in 8 µl/mg of homogenization buffer using a Potter (15 pulses). Immediately after, 0.08 µl/mg of BCD buffer (homogenization buffer, 10% Igepal, 5% sodium deoxycholate, 1% SDS, 250 mM CHAPS) were added to each sample. Samples were vortexed and kept in ice for 30 min, centrifuged for 10 min at 20,000*g* (4°C), and supernatants kept. Protein content was determined using a Bio-Rad DC Protein Assay Kit with BSA as standard. Samples were then diluted in homogenization buffer until reaching a concentration of 4 mg protein/ml, and aliquoted. Commercial Laemmli buffer (95% v/v) and β-mercaptoethanol (5% v/v) were added to each sample. Finally, all the samples were vortexed, heated at 95°C for 5 min and kept at −70°C until Western blot experiments were performed.

### Western Blot

Western blot was performed as previously described (Ibarra-Lecue et al., 2018) with minor modifications. Samples were heated (95°C), loaded (30 μg) and submitted to SDS-PAGE onto polyacrylamide gel (12%). Nitrocellulose membranes were blocked (5% non-fat dry-milk or/and 0.5% BSA) in TBS buffer followed by overnight incubation with primary antibodies (4°C). Specific antibodies against Akt, phospho-Akt(Ser473), GSK3α/β, phospho-GSK3α/β(Ser21/9), S6, phospho-S6(Ser235/236), and β-actin were used. Dilutions of primary antibody have been previously evaluated for a signal within the linear range of detection (Ibarra-Lecue et al., 2018). Incubation with fluorescent anti-IgG secondary antibodies was performed at room temperature (1 h) (further details about antibodies and dilutions are in **Supplementary Table 3**). Immunoreactivity was quantified using an Odyssey Infrared Imaging System (LI-COR Biosciences, Lincoln, NE, USA). Representative images of immunoblots can be found in **Supplementary Figure 1** (human) and **Supplementary Figure 2** (rat).

### Data Analysis and Statistical Procedures

Data were analyzed with GraphPad Prism™ version 7.0 (GraphPad Software, San Diego, CA, USA) and InVivoStat free software. Two-group comparisons (C vs SZ) were made by unpaired Student’s *t*-test. Multiple groups’ comparisons (C, AP-F, AP-T) were studied by one-way analyses of variance (ANOVA), followed by Bonferroni’s *post hoc* analyses. Linear regression analysis was used to assess the contribution of age, PMI, and storage time to the protein immunoreactivity values. When significant differences were observed between groups, subsequent analyses of covariance (ANCOVA) were performed to discard the effect of these potential confounding variables on the observed differences. Statistical significance was set at *p* < 0.05. Immunodensitometric values of the different target proteins were normalized to the intra-assay values obtained with anti-β-actin antibody and expressed as mean ± SEM of the percentages of an inter-assay normalization sample included in every experiment. Inter-assay normalization sample was a total homogenate from pooled brain cortical tissue of six subjects (3 C and 3 SZ) or six rats. This sample was included in every gel and β-actin corrected values of the target proteins of all the study samples are referred as a percentage from this inter-assay normalization sample. Each sample was analyzed at least in two independent experiments. All data were subjected to a Grubbs’s test to determine possible outlier values. The detected outliers were rejected for the statistical analysis.

## Results

### The mTORC1 Pathway is Dysregulated in Schizophrenia

**Figure 1:**
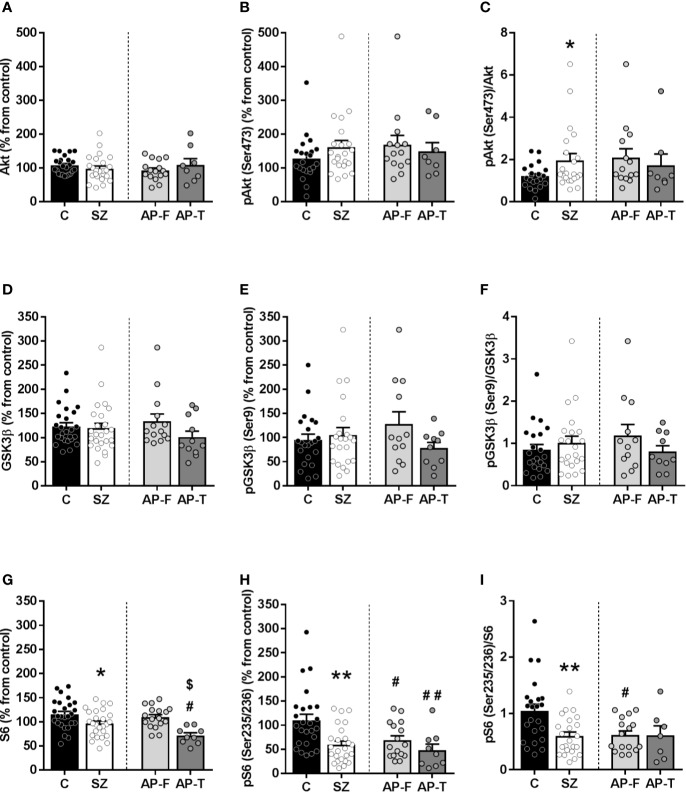
Protein expression of signaling proteins in postmortem prefrontal cortex (PFC) from subjects with schizophrenia (SZ) [antipsychotic-treated (AP-T), or drug-free (AP-F)] and matched controls (C). Total Akt **(A)** and phospho-Akt(Ser473) protein levels **(B)**. **(C)** Phospho-Akt(Ser473)/Akt ratio. Total GSK3β **(D)** and phospho-GSK3β(Ser9) protein levels **(E)**. **(F)** Phospho-GSK3β(Ser9)/GSK3β ratio. Total S6 **(G)** and phospho-S6(Ser235/236) protein levels **(H)**. **(I)** Phospho-S6(Ser235/236)/S6 ratio. *p < 0.05 and **p < 0.01 *vs* controls; unpaired Student's *t* test analysis. **^#^***p* < 0.05 and **^##^***p* < 0.005 *vs* controls and **^$^***p* < 0.05 *vs* AP-F; one-way analysis of variance (ANOVA) followed by Bonferroni *post-hoc* test. Data represent the means ± standard errors of the mean (SEM) referred as a percentage of the inter-assay normalization sample.

In the human PFC of schizophrenic subjects, Akt levels did not differ from that in matched controls (controls (C): 108% ± 5%; schizophrenic subjects (SZ): 98% ± 8%) (**Figure 1A**). When subjects were divided regarding antipsychotic presence/absence in blood, Akt immunodensity did not show significant differences between AP-F (93% ± 7%) and AP-T subjects (109% ± 18%) (**Figure 1A**). Active phosphorylated form of Akt (phospho-Akt(Ser473)) did not differ between SZ (161% ± 19%), with or without antipsychotic treatment (AP-F: 168% ± 27%; AP-T: 149% ± 25%) and matched controls (C: 128% ± 14%) (**Figure 1B**). After calculating the phospho-Akt(Ser473)/Akt ratio for each subject, a significant increase in the proportion of the active/phosphorylated form was found in the SZ group (*p* < 0.05) compared to matched controls, while no differences were found between AP-F and AP-T subjects (**Figure 1C**).

The immunodensity of GSK3β was found unaltered in schizophrenia (C: 122% ± 8%; SZ: 119% ± 10%) (**Figure 1D**), and the antipsychotic treatment did not modify significantly the content of the protein (AP-F: 134% ± 14%; AP-T: 100% ± 12%). The inactive/phosphorylated form (phospho-GSK3β(Ser9)) did not show differences among groups (SZ: 105% ± 15%; C: 95% ± 12%; AP-F: 128% ± 26%; AP-T: 78% ± 12%) (**Figure 1E**). In the same way, no significant differences were found in the phospho-GSK3β (Ser9)/GSK3β ratio (**Figure 1F**).

Total S6 density was significantly decreased (*p* < 0.05) in SZ compared to matched controls (SZ: 97% ± 5%; C: 115% ± 6%) (**Figure 1G**). When schizophrenia samples were divided regarding the presence of antipsychotic drugs in blood at the time of death, this decrease was only statistically significant (*p* < 0.05) in AP-T (71% ± 5%) when comparing with AP-F (109% ± 5%) and with controls (**Figure 1G**).

The density of the active phosphorylated form of the S6, phospho-S6(Ser235/236), showed a significant decrease (*p* < 0.05) in both AP-F and AP-T subjects compared to matched controls (C: 110% ± 12%; SZ: 59% ± 7%; AP-F: 68% ± 9%; AP-T: 48% ± 12%) (**Figure 1H**). Consequently, the phospho-S6(Ser235/236)/S6 ratio showed a significant decrease in the whole population of schizophrenia samples (*p* < 0.05). This decrease was also evident in both AP-F and AP-T groups, although only in the AP-F subjects this reduction was significant (*p* < 0.05) when compared to controls (**Figure 1I**).

In order to ensure whether the differences observed in protein levels between schizophrenia and control subjects were influenced by confounding variables, the effect of age, PMI and storage time was assessed.

The phosphorylated form of S6 was significantly influenced by age but not by the PMI or storage time. Pearson’s correlation analyses showed a significant negative correlation between phospho-S6(Ser235/236) levels and age at death in both controls (*p* < 0.05, r = −0.39) and subjects with schizophrenia (*p* < 0.05, r = −0.43) (**Supplementary Figure 3**). Linear regression analyses showed no significant differences in the slopes between groups. In an effort to control the confounding influence of this variable, ANCOVA analyses were performed with protein level values as the dependent variable, and age as covariate. Consistent with the previous findings, statistically significant differences between schizophrenia and control subjects were still observed [F(1,47) = 14.26, p = 0.0004].

### Haloperidol and Clozapine, but not Risperidone, Modulate Akt Signaling in Rat Brain Cortex

**Figure 2:**
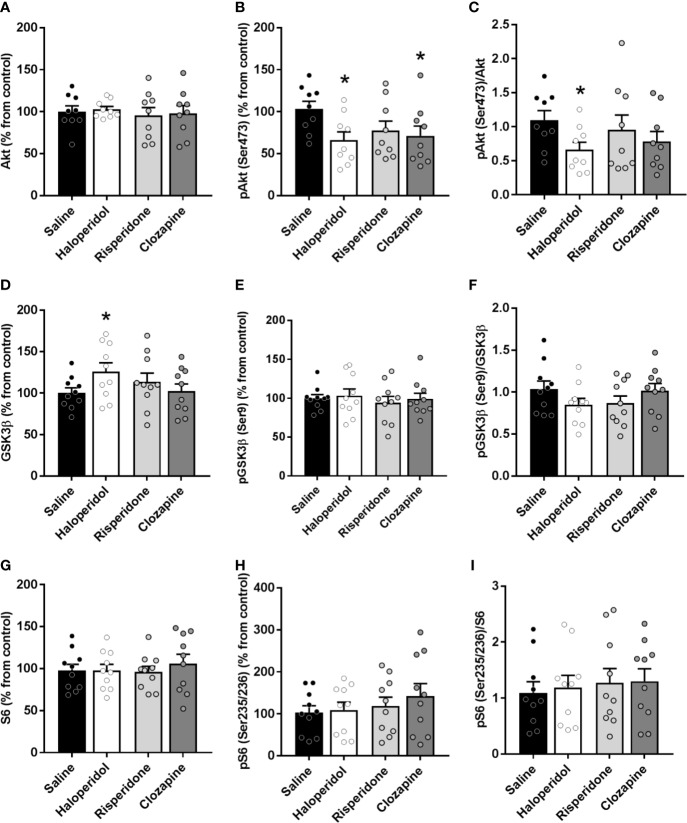
Protein expression of signaling proteins in cortical tissue from rats treated with saline, haloperidol, risperidone, or clozapine. Total Akt **(A)** and phospho-Akt(Ser473) levels **(B)**. **(C)** Phospho-Akt(Ser473)/Akt ratio. Total GSK3β **(D)** and phospho-GSK3β(Ser9) levels **(E)**. **(F)** Phospho-GSK3β(Ser9)/GSK3β ratio. Total S6 **(G)** and phospho-S6(Ser235/236) levels **(H)**. **(I)** Phospho-S6(Ser235/236)/S6 ratio. *p < 0.05 *vs* controls; one-way ANOVA followed by Bonferroni *post-hoc* test. Data represent the means ± SEM, referred as a percentage of the inter-assay normalization sample.

Both haloperidol and clozapine induced a significant decrease (*p* < 0.05) in the active form phospho-Akt(Ser473) compared to saline controls (saline: 103% ± 9%; haloperidol: 66% ± 9%; risperidone: 77% ± 11%; clozapine: 71% ± 11%) while no changes were found in Akt total form (**Figures 2A, B**). A significant decrease in the phospho-Akt(Ser473)/Akt ratio was also observed in brain cortex of rats treated with haloperidol (*p* < 0.05) (**Figure 2C**).

Conversely, haloperidol induced a significant increase (*p* < 0.05) in the levels of total GSK3β (saline: 100% ± 6%; haloperidol: 126% ± 10%; risperidone: 113% ± 10%; clozapine: 102% ± 8%) (**Figure 2D**), while no changes in the phosphorylated form or the ratio were observed (**Figures 2E, F**). Clozapine did not induce any significant change in GSK3β (**Figure 2D**) or pGSK3β (Ser9) proteins (**Figure 2E**). Risperidone was not able to modulate the levels of any of the proteins evaluated. No changes were observed neither in S6 (**Figure 2G**), nor in phospho-S6 (Ser235/236) (**Figure 2H**), nor in the ratio (**Figure 2I**) in the brain cortex of rats treated with any of the antipsychotic drugs.

## Discussion

The main finding of our study is the strong decrease in the expression and phosphorylation of S6 in the PFC of subjects with schizophrenia. To our knowledge, this is the first report of a dysfunction in this downstream effector of mTORC1 in human brain.

**Figure 3:**
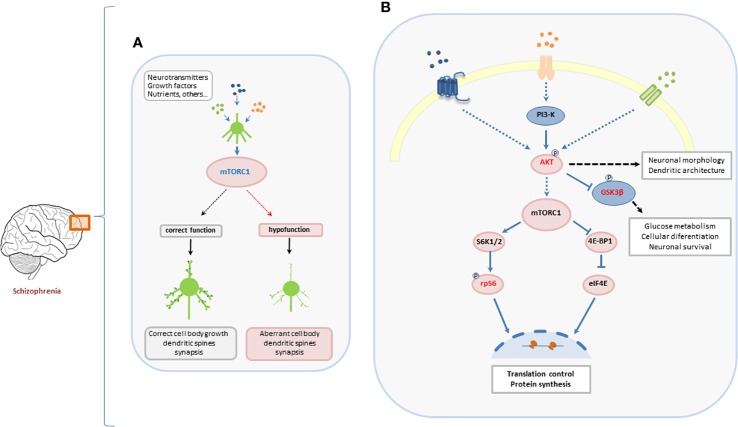
Suggested role of mTORC1-dependent signaling pathway in the development of neurons and its implication in aberrant development of PFC in schizophrenia. **(A)** Neurotransmitters, growth factors, nutrients, and other environmental inputs coordinately activate mTORC1 leading to a correct normal neuronal development. Hypofunction of mTORC1 induces a dysregulation of the neuronal development that may be involved in the development of psychiatric disorders. **(B)** The canonical Akt/mTORC1/S6 pathway. The two main targets of mTORC1, S6K, and 4E-BP1, are involved in the translational machinery for protein synthesis. Once active, S6K regulates the activity of the ribosomal protein S6 by phosphorylating it at different sites. The phosphorylation of S6 controls translation and regulates protein synthesis in the central nervous system (CNS).

Both environmental factors and extracellular stimuli previously implicated in the development of schizophrenia are known to control the mTOR cascade. The control of protein synthesis carried out by the activation of ribosomal protein S6 may underlie aspects of neurodevelopment and synaptic plasticity (Jaworski and Sheng, 2006; Ruvinsky and Meyuhas, 2006; Tang et al., 2014). This hypothesis is supported by data suggesting that dysfunctions in mTORC1/S6 pathway lead to the inhibition of oligodendrocyte precursor cells proliferation and maturation and the subsequently hypomyelination (Liu et al., 2014; Maas et al., 2017). In this context, the present results suggest a hypofunction of mTORC1 pathway in schizophrenia. This hypofunction might contribute to dysfunctions in brain connectivity and the development of the neurobiological manifestations of schizophrenia (**Figure 3**).

One of the upstream regulators of mTORC1 is Akt, an intermediator of different cellular pathways that acts in response to different extracellular stimuli. In neurons, the activation of Akt induces the phosphorylation of different substrates that, thereby, regulates a wide variety of processes such as neuronal development, morphogenesis, dendritic development, and synaptic plasticity. Growing evidence indicates that the mTORC1/S6K pathway mediates potent negative feedback loops that restrain upstream signaling. In this way, inhibition of mTORC1/S6K by specific drugs suppresses these feedback loops and causes compensatory over-activation of upstream signaling proteins, including Akt (Rozengurt et al., 2014). Thus, it has been reported that an outstanding consequence of mTORC1/S6K inhibition is the notable increase in Akt phosphorylation (O’Reilly et al., 2006; Lane and Breuleux, 2009; Rozengurt et al., 2014). Results of the present study showed a significant increase in the ratio phospho-Akt(Ser473)/Akt in cortical tissue of SZs compared with their matched controls. These data, together with the observed decrease in S6 function, point to a suppression of the feedback loops that control the PI3K/Akt/mTOR signaling.

To date, studies evaluating levels of Akt in human postmortem brains of SZs show divergent results. The first study analyzed frontal cortex samples obtained from two different brain banks (without specifying the anatomical subregions evaluated) and reported decreased Akt protein levels (Emamian et al., 2004). Another study (Zhao et al., 2006) found a decrease in the ratio phosphorylated/total Akt in the PFC (BA46) of patients with schizophrenia. The study of Ide assessed the protein levels of Akt in frontal cortex (BA9) from the samples from the Stanley foundation (Ide et al., 2006), the same resource as one of the two used in Emamian´s study, but failed to find differences. In this study, another different cohort of samples was used (frontal cortex BA9) and no differences were found between both groups. Another study (Hino et al., 2016) found an increase in phosphorylated Akt, though the ratio of phospho/total Akt was not significantly different in postmortem tissue. On the contrary, the most recent study evaluating Akt in schizophrenia found a decrease in phospho/total Akt ratio in DLPFC (Chadha and Meador-Woodruff, 2020). These controversial results could be explained by the different cortical subregions evaluated, the different antibodies used and, mainly, by confounding factors such as age, sex, PMI, or antipsychotic treatment that have not been controlled in the vast majority of studies.

GSK3 activity is known to influence neural progenitor cell proliferation and differentiation, as well as efficient neurotransmission in differentiated adult neurons (Cole, 2012). GSK3 is indeed an important mediator of dopamine action *in vivo*, and modulation of the Akt/GSK3 pathway is thought to be relevant in dopamine-related disorders (Beaulieu et al., 2004). Recent studies have also suggested a role for this pathway in synaptic modulation and plasticity during the adult life (Hall et al., 2000). Moreover, antipsychotics regulate GSK3 (Beaulieu, 2007) thus supporting the postulate that abnormalities in dopaminergic and/or serotonergic systems in schizophrenia could be associated with abnormal GSK3 control (Jope and Roh, 2006). However, the direct association of GSK3 with psychiatric disorders is less clear, and studies addressing the association between GSK3 and schizophrenia show conflicting results. Previous studies have found both reduced (Kozlovsky et al., 2001; Emamian et al., 2004; Amar et al., 2008) and unchanged (Nadri et al., 2002; Ide et al., 2006) protein expression of GSK3β in postmortem PFC from subjects with schizophrenia. In this context, the different brain regions analyzed may have a putative influence in the contradictory results. Under our experimental conditions, we failed to find significant differences in the protein expression of GSK3β or its phosphorylated form phospho-GSK3β(Ser9) between SZs and matched controls.

An increasing number of studies have used S6 phosphorylation as a readout of mTOR activity and as a marker for neuronal activation in the context of synaptic plasticity or in response to therapeutic agents (Bonito-Oliva et al., 2013; Bowling et al., 2014; Biever et al., 2015; Henry et al., 2018). It has been proposed that the phosphorylation of S6 could participate in the positive regulation of global cellular translation (Ruvinsky and Meyuhas, 2006) and in dendrite development (Jaworski and Sheng, 2006; Tang et al., 2014). Thus, the inhibition of mTOR induces a substantial reduction in the number of dendritic branches and arbor shrinkage in neurons (Jaworski and Sheng, 2006). On the contrary, excessive mTORC1 activation is linked to increased dendritic spine density and aberrant synaptic pruning in autism spectrum disorders (Tang et al., 2014).

Interestingly, alterations in mTOR signaling pathway have been described in rodent models used for the study of mental disorders. In this context, a single administration of psychotomimetic drugs, such as ketamine, MK-801 and scopolamine, seems to activate mTOR signaling pathway in the rat brain cortex (Yoon et al., 2008; Li et al., 2010; Voleti et al., 2013). Whereas these drugs are being studied in relation with their fast antidepressant action, mTOR pathway activation seems to be necessary to reestablish synaptic deficits that result from exposure to stress in animal models (Duman et al., 2016). Developmental models of schizophrenia, such as repeated phencyclidine (PCP) treatment during neonatal period and post-weaning isolation have shown an increased phosphorylation of S6 specifically in the rat PFC, and not in striatum (Meffre et al., 2012). Furthermore, early postnatal treatment with the NMDA receptor antagonist MK-801 affects mTOR/p70S6K-related pathways in the frontal cortex of the adult rat brain (Kim et al., 2010). Chronic treatment with ketamine leads to downregulation of mTOR protein expression in both PFC and hippocampus of rats (Xie et al., 2020). Interestingly, chronic THC exposure during adolescence induces schizophrenia-like behaviors and decreases mTOR-p70S6K signaling pathway in the PFC of adult rat brains (Renard et al., 2016).

To our knowledge, the present is the first study exploring this mTORC1 downstream effector in postmortem PFC tissue of subjects with schizophrenia. The phosphorylation ratio of S6 is strongly reduced in schizophrenia and this reduction occurs in both AP-F and AP-T subjects suggesting that phospho-S6(Ser235/236) expression is dysregulated as a consequence of the disorder itself. Even so, our results are also in accordance with a study that found a reduction in ribosomal proteins and in protein synthesis in schizophrenia (English et al., 2015). In this study, authors found reduced cell area, ribosomal protein expression, and rates of protein synthesis in schizophrenia patient-derived olfactory cells. Notably, they reported a striking uniform decreased expression of 17 ribosomal proteins, including S6, although they did not evaluate the active/phosphorylated form of this ribosomal protein. Consistent with our findings, a recently published study also provides evidence that proteins associated with the AKT-mTOR signaling cascade are downregulated in schizophrenia DLPFC postmortem tissue (Chadha and Meador-Woodruff, 2020).

However, there are some limitations to consider in interpreting results from this study. The levels and phosphorylation status of these proteins may be conditioned by numerous factors that affect postmortem samples, such as sex, age, the storage time, or PMI. These variables have been taken into account in our study by matching each subject with schizophrenia with a control of the same sex and with similar age, and PMI and were performed correlation analyses to assess associations between protein expression/phosphorylation and age, PMI, and storage time. However, there are additional confounders such as exercise, diet, or tobacco smoking that we are not able to control. In addition, and unfortunately, the size of the groups hinders to extract conclusions regarding gender effects.

On the other hand, the treatment-controlled design allows us to study the putative influence of chronic antipsychotic drugs on protein density and phosphorylation.

Studies about the effects of antipsychotics in Akt/mTOR pathway are contradictory showing both increases, decreases, or no changes in the expression and functional levels of Akt, GSK3 and/or S6 (Alimohamad et al., 2005; Li et al., 2007; Bonito-Oliva et al., 2013; Pereira et al., 2013; Bowling et al., 2014; Mas et al., 2016).

In our study, both chronic haloperidol and clozapine decreased the phospho-(Ser473)Akt levels in rats, but only chronic haloperidol showed a significant decrease in the phospho-(Ser473)Akt/Akt ratio. It has been proposed that depending on its duration, activation of D2 receptors (D2R) differentially regulates Akt-dependent pathways. In this context, acute activation of D2R triggers an inactivation of Akt (Beaulieu et al., 2004). In line with this, acute treatments with haloperidol and clozapine, both exerting a D2R antagonist effect, increase phospho-(Ser473)Akt (Roh et al., 2007). In cell cultures, stimulation of D2R rapidly activates Akt signaling (Mannoury La Cour et al., 2011). By contrast, prolonged stimulation of D2R decreases Akt phosphorylation (Beaulieu et al., 2005). Taking all these data into account, continuous activation of D2R may be triggering a decrease in the tonic phospho-(Ser473)Akt levels observed in chronic haloperidol treated rats. Nevertheless, differences in the effect among antipsychotics due to the affinity for other receptors cannot be ruled out (Beaulieu, 2012).

In this study, chronic haloperidol was the only one exerting an effect over GSK3β, triggering a significant increase in total GSK3β expression in rat cortex. Although the literature is scarce, both acute haloperidol and clozapine seem to decrease the phosphorylation of GSK3β at Ser9 *in vitro* (Takaki et al., 2018). On the contrary, a study carried out in mice reported an increased in the phosphorylation status after acute clozapine and risperidone, with no changes with haloperidol (Li et al., 2007). Similar to our results, another study showed that both sub-chronic and chronic administration of haloperidol resulted in an increased expression of total GSK3 (Alimohamad et al., 2005).

It has been described that acute haloperidol increases rpS6 phosphorylation in rodent striatal neurons (Bonito-Oliva et al., 2013; Bowling et al., 2014). Moreover, an increase or a decrease in rpS6 phosphorylation has been reported in the striatum after acute haloperidol and risperidone treatment depending on the strain of the mice used (Mas et al., 2016). On the other hand, acute clozapine seems to decrease the phosphorylation of the S6K in both cortex and striatum (Pereira et al., 2013). However, all these studies have been carried out under acute treatments. Our results suggest no changes in the levels of rpS6 phosphorylation in the cortex of rats chronically treated with antipsychotics. Divergent results in rpS6 phosphorylation among studies could be due to differences in doses and modes of administration of antipsychotics, in brain regions used, and in the strains of animals used (Mas et al., 2016). These results suggest that the effects of antipsychotics on the Akt/mTOR pathway are different in schizophrenia patients and rodents, both because of species related differences, and because animals used here lack the pathophysiological substrates of schizophrenia. In fact, it is possible that antipsychotics in humans could have different effects in the normal brain and in schizophrenia-related neuropathology (Goff et al., 2017).

As previously stated, S6 participates in the positive regulation of global cellular translation (Ruvinsky and Meyuhas, 2006). Our findings, together with the reduced cell area, ribosomal protein expression and rates of protein synthesis previously reported (English et al., 2015), are consistent with the reduction of the neuronal body size (Rajkowska et al., 1998; Chana et al., 2003) and of the whole-brain volume in schizophrenia (Harrison and Weinberger, 2005; Cannon et al., 2015). Dysfunctions in mTORC1/S6 pathway inhibits oligodendrocyte proliferation and maturation (Liu et al., 2014; Maas et al., 2017), contributing to an alteration in white matter integrity in schizophrenia (Flynn et al., 2003; Schoonover et al., 2019). On the other hand, the inhibition of mTORC1-dependent signaling involves a substantial reduction in the number of dendritic branches and arbor shrinkage in developing neurons (Jaworski and Sheng, 2006; Kwon et al., 2006).

The downregulation of Akt/mTOR signaling proteins in postmortem PFC of SZs (Chadha and Meador-Woodruff, 2020), together with postmortem studies showing that spine density is reduced in the cortex of schizophrenia patients (Glantz and Lewis, 2000; Konopaske et al., 2014) further support our results. Altogether, these alterations in protein synthesis and dendritic architecture would finally contribute to dysfunctions in synaptic connectivity that underlay clinical manifestations of schizophrenia.

## Data Availability Statement

All datasets generated for this study are included in the article/**Supplementary Material**.

## Ethics Statement

The animal study was reviewed and approved by the Ethic Committee for Animal Welfare of the University of the Basque Country, UPV/EHU (CEBA 188/2011).

## Author Contributions

LU and LC designed experiments. BM, LC, and JM obtained and classified postmortem human brain samples. Western blot assays were performed by II-L and RD-A. II-L and LU analyzed data and wrote the manuscript. All the contributors critically revised and approved the final version of the manuscript.

## Funding

This study was funded by the Instituto de Salud Carlos III, FEDER (PI13/01529) and the Basque Government (IT211-19). II-L is recipient of a Predoctoral Fellowship from the Basque Government.

## Conflict of Interest

The authors declare that the research was conducted in the absence of any commercial or financial relationships that could be construed as a potential conflict of interest.
