# Supplementary material for: Ribosomal Protein S6 Hypofunction in Postmortem Human Brain Links mTORC1-Dependent Signaling and Schizophrenia
Source: Front Pharmacol. 2020 Mar 24;11:344. doi: 10.3389/fphar.2020.00344 (PMC7105616; doi:10.3389/fphar.2020.00344)
Supplement: Supplementary file 1 [file Image_1.pdf]

Supplementary Figure 1

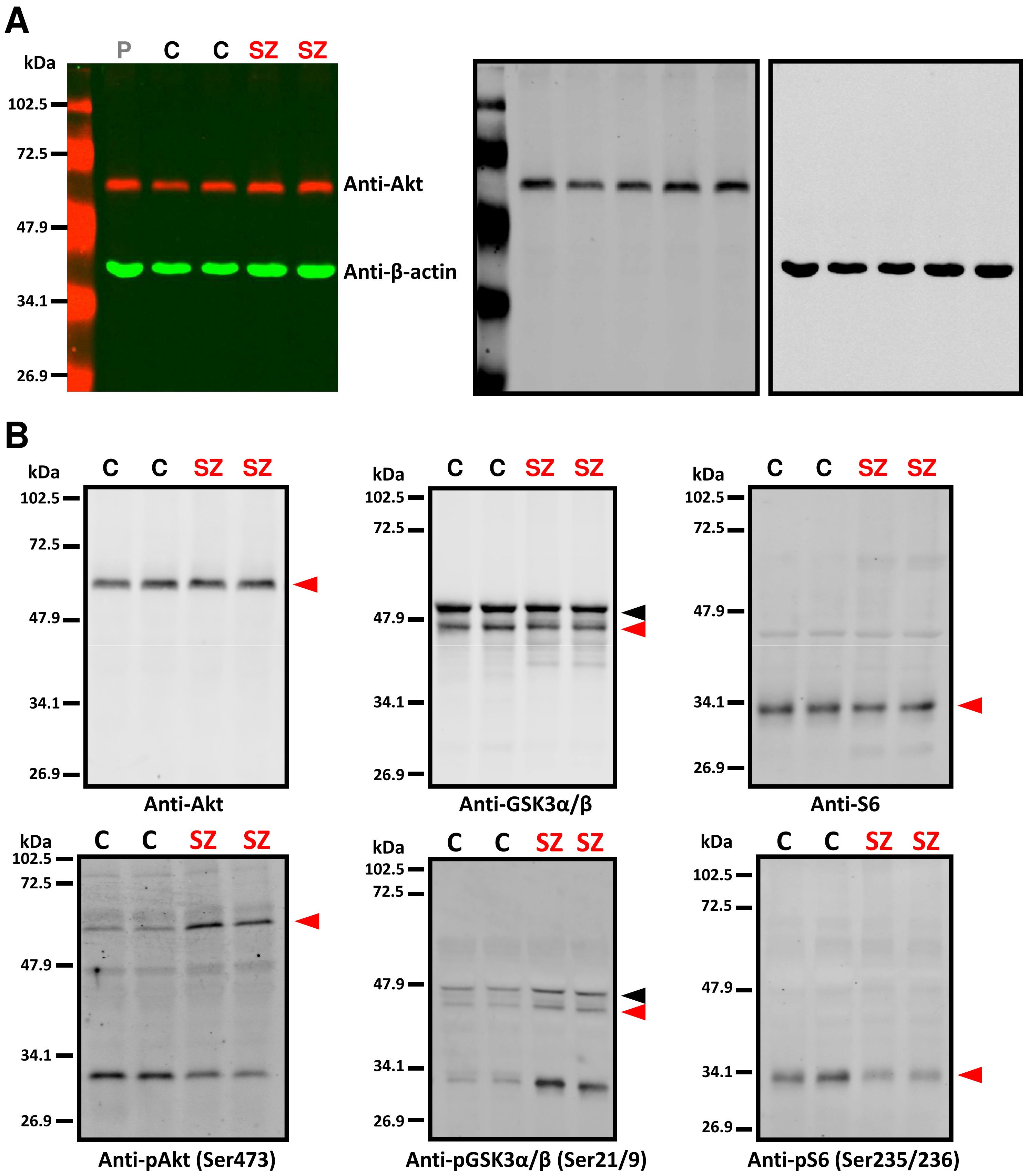

Figure S1. Representative images of immunoblots carried out in human PFC in the study. **A.** Image of 700 and 800nm channels visualized overlaid and separately. **B.** Images of 700nm channel, where the six target proteins were visualized in the experiments. Red arrows show the bands that were analyzed. Black arrows show the α subunit of GSK3, that was not analyzed in this study. C = control; SZ = schizophrenia; P = pool/inter-experimental control; kDa = kDaltons.
