# Supplementary material for: Ribosomal Protein S6 Hypofunction in Postmortem Human Brain Links mTORC1-Dependent Signaling and Schizophrenia
Source: Front Pharmacol. 2020 Mar 24;11:344. doi: 10.3389/fphar.2020.00344 (PMC7105616; doi:10.3389/fphar.2020.00344)
Supplement: Supplementary file 2 [file Image_2.pdf]

Supplementary Figure 2

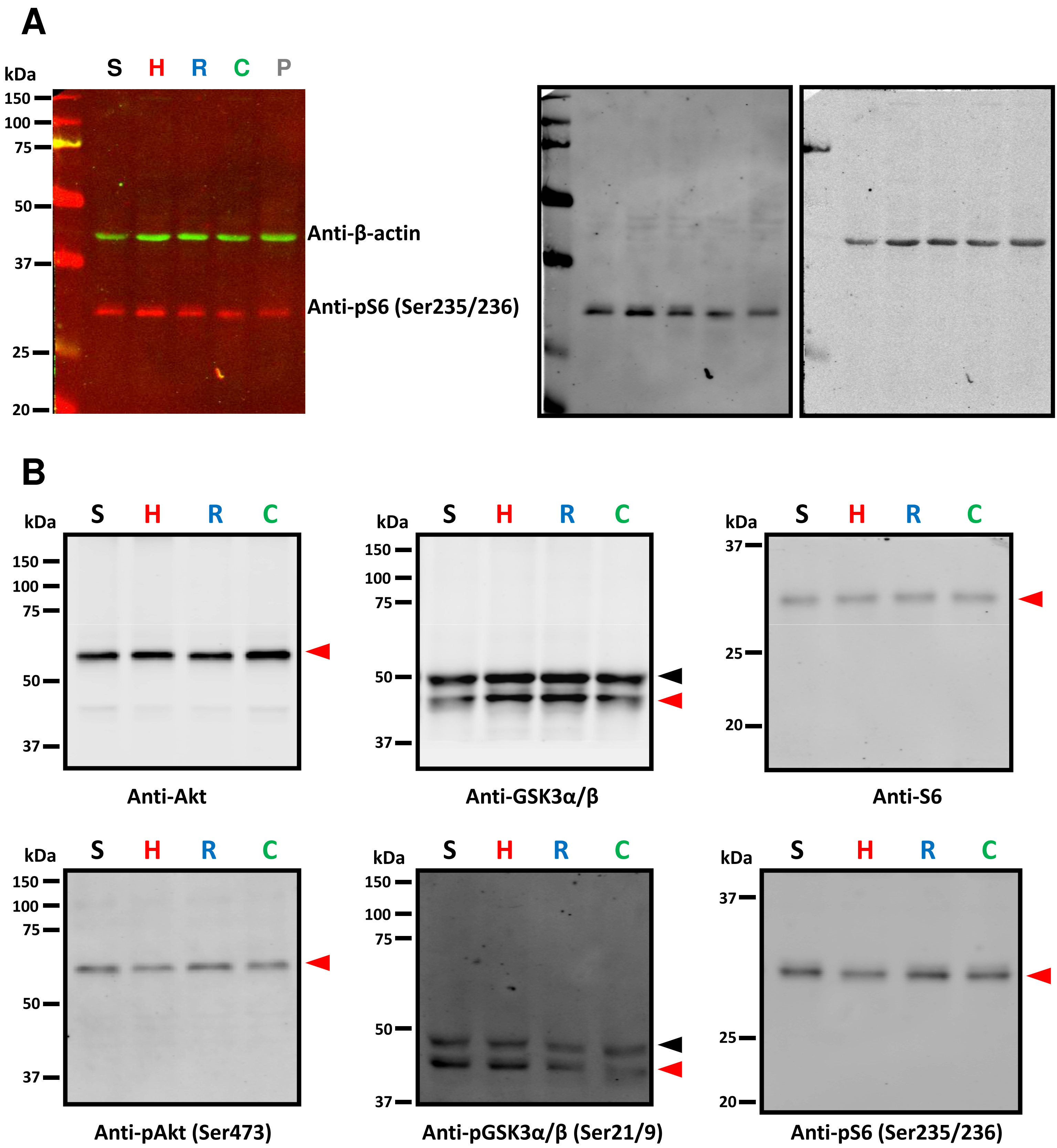

Figure S2. Representative images of immunoblots carried out in rat cortex in the study.
A. Image of 700 and 800nm channels visualized overlaid and separately. **B.** Images of 700nm channel, where the six target proteins were visualized in the experiments. Red arrows show the bands that were analyzed. Black arrows show the α subunit of GSK3, that was not analyzed in this study. S = saline; H = haloperidol; R = risperidone; C = clozapine; P = pool/inter-experimental control; kDa = kDaltons.
