# Supplementary material for: Ribosomal Protein S6 Hypofunction in Postmortem Human Brain Links mTORC1-Dependent Signaling and Schizophrenia
Source: Front Pharmacol. 2020 Mar 24;11:344. doi: 10.3389/fphar.2020.00344 (PMC7105616; doi:10.3389/fphar.2020.00344)
Supplement: Supplementary file 3 [file Image_3.pdf]

**Supplementary Figure 3**

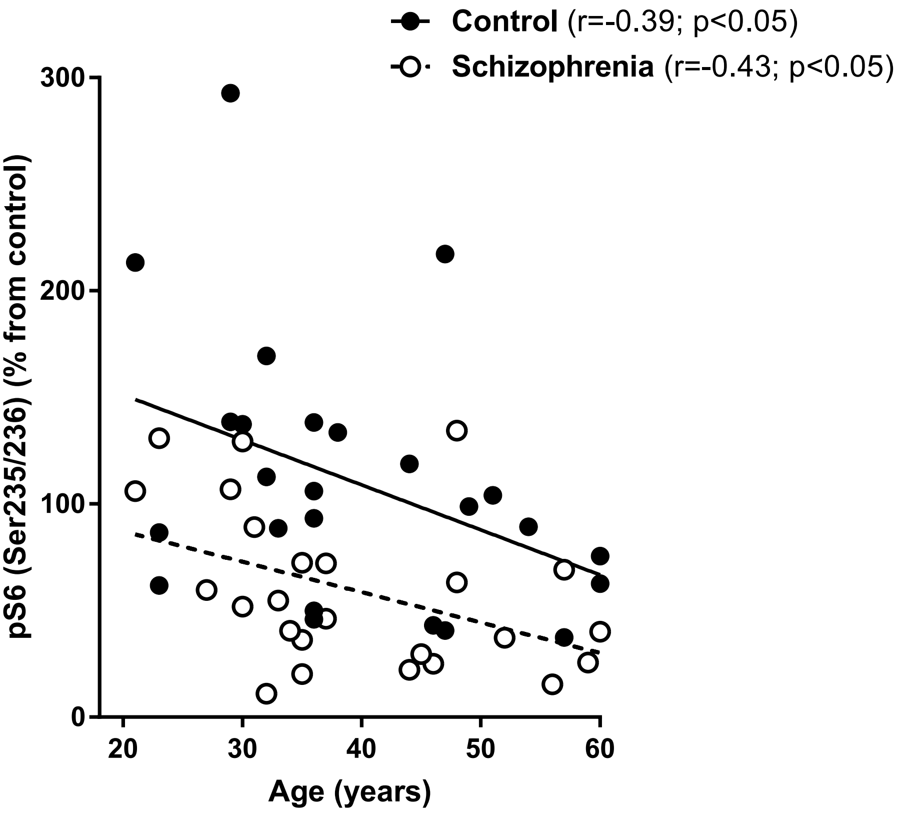


**Figure S3. Correlations between pS6 protein levels and age at death in controls (n=25) and schizophrenic subjects (n=25). Linear regressions, r and p values for Pearson's correlations are shown.**
