# Supplementary material for: Ribosomal Protein S6 Hypofunction in Postmortem Human Brain Links mTORC1-Dependent Signaling and Schizophrenia
Source: Front Pharmacol. 2020 Mar 24;11:344. doi: 10.3389/fphar.2020.00344 (PMC7105616; doi:10.3389/fphar.2020.00344)
Supplement: Supplementary file 4 [file Table_1.pdf]

Supplementary Table 1. Demographic characteristics, *postmortem* interval (PMI), cause of death and toxicological study of antipsychotic-free schizophrenic subjects (SCH) and matched control subjects (C).

Case	Gender	Age (years)	PMI (hours)	Storage time (months)	Cause of death	Psychiatric diagnosis	APs in blood
SCH1	M	21	24	175	Suicide/Jumping	Schizophrenia	-
C1	M	21	30	135	Accident/Traffic	Control	-
SCH2	M	30	51	144	Suicide/Jumping	Schizophrenia	-
C2	M	29	18	32	Accident/Falling	Control	-
SCH3	M	29	6	79	Suicide/Asphyxia	Schizophrenia	-
C3	M	29	36	137	Accident/Traffic	Control	-
SCH4	M	31	14	78	Suicide/Jumping	Schizophrenia	-
C4	M	32	28	78	Accident/Traffic	Control	-
SCH5	M	48	20	74	Suicide/Train	Schizophrenia	-
C5	M	47	18	94	Natural/CRF	Control	-
SCH6	M	33	14	64	Suicide/Hanging	Schizophrenia	-
C6	M	33	4	52	Accident/Traffic	Control	-
SCH7	M	45	3	60	Suicide/Gun	Schizophrenia	-
C7	M	44	21	46	Accident/Traffic	Control	-
SCH8	M	27	24	60	Suicide/Gun	Schizophrenia	-
C8	M	28	30	132	Accident/Traffic	Control	-
SCH9	F	37	58	44	Suicide/Overdose	Schizophrenia	-
C9	F	36	38	168	Homicide/Knife	Control	-
SCH10	M	46	22	35	Suicide/Jumping	Schizophrenia	-
C10	M	46	24	23	Natural/MI	Control	-
SCH11	F	37	26	9	Suicide/Jumping	Schizophrenia	-
C11	F	38	22	6	Accident/Traffic	Control	-
SCH12	M	48	11	9	Suicide/Jumping	Schizophrenia	-
C12	M	49	8	3	Natural/CRF	Control	-
SCH13	M	35	5	10	Suicide/Hanging	Schizophrenia	-
C13	M	38	33	165	Accident/Traffic	Control	-
SCH14	F	59	9	15	Natural/CRF	Schizophrenia	-
C14	F	58	20	136	Natural/CRF	Control	-
SCH15	M	45	18	19	Suicide/Jumping	Schizophrenia	-
C15	M	47	15	4	Accident/traffic	Control	-
SCH16	M	34	15	21	Natural/CRF	Schizophrenia	-
C16	M	36	48	15	Natural/CRF	Control	-
SCH17	M	52	7	26	Suicide/Jumping	Schizophrenia	-
C17	M	51	13	6	Accident/Traffic	Control	-
Sch AP-F	14M/3F	38.6±2	19.2±3	54.2±11			
C	14M/3F	40.3±3	23.8±3	48.7±13			

F (Female), M (Male), CRF (cardiorespiratory failure), MI (myocardial infarct), APs (antipsychotics). Mean ± S.E.M.
