# Supplementary material for: Ribosomal Protein S6 Hypofunction in Postmortem Human Brain Links mTORC1-Dependent Signaling and Schizophrenia
Source: Front Pharmacol. 2020 Mar 24;11:344. doi: 10.3389/fphar.2020.00344 (PMC7105616; doi:10.3389/fphar.2020.00344)
Supplement: Supplementary file 5 [file Table_2.pdf]

Supplementary Table 2. Demographic characteristics, *postmortem* interval (PMI), cause of death and toxicological study of antipsychotic treated schizophrenic subjects (SCH) and matched control subjects (C).

Case	Gender	Age (years)	PMI (hours)	Storage time (months)	Cause of death	Psychiatric diagnosis	APs in blood
SCH18	M	44	7	84	Natural/CRF	Schizophrenia	CTP
C18	M	44	23	4	Accident/Traffic	Control	-
SCH19	M	30	18	81	Suicide/Jumping	Schizophrenia	OLZ
C19	M	30	11	82	Accident/Electrocution	Control	-
SCH20	M	32	8	77	Suicide/Hanging	Schizophrenia	QTP
C20	M	32	20	135	Accident/Crushing	Control	-
SCH21	M	23	16	72	Suicide/Jumping	Schizophrenia	SLP
C21	M	23	17	45	Accident/Electrocution	Control	-
SCH22	M	35	3	72	Suicide/Jumping	Schizophrenia	QTP
C22	M	36	23	4	Accident/Crushing	Control	-
SCH23	M	35	11	24	Natural/CRF	Schizophrenia	CLZ
C23	M	36	18	94	Accident/Traffic	Control	-
SCH24	F	60	23	14	Natural/Peritonitis	Schizophrenia	ASLP/CLZ
C24	F	60	48	20	Natural/CH	Control	-
SCH25	M	56	12	19	Natural/CRF	Schizophrenia	OLZ/CLT
C25	M	54	16	10	Accident/Traffic	Control	-
SCH26	F	30	17	24	Suicide/Jumping	Schizophrenia	HLP
C26	F	30	18	17	Accident/Traffic	Control	-
SCH27	M	57	19	24	Suicide/Train	Schizophrenia	QTP
C27	F	57	14	21	Natural/CRF	Control	-
SCH28	M	26	39	19	Suicide/Jumping	Schizophrenia	OLZ
C28	M	25	18	14	Accident/Fire	Control	-
Sch AP-T	9M/2F	38.9±3	16.1±2	46.3±9			
C	8M/3F	36.6±3	20.4±2	41.4±1			

F (Female), M (Male), CRF (cardiorespiratory failure), CH (cerebral hemorrhage). Antipsychotics (APs) in blood are coded as ASLP (amisulpiride), BIP (biperiden), CLZ (clozapine), CTP (clotiapine), HLP (haloperidol), OLZ (olanzapine), QTP (quetiapine), SLP (sulpiride). Mean ± S.E.M.
