# Supplementary material for: Ribosomal Protein S6 Hypofunction in Postmortem Human Brain Links mTORC1-Dependent Signaling and Schizophrenia
Source: Front Pharmacol. 2020 Mar 24;11:344. doi: 10.3389/fphar.2020.00344 (PMC7105616; doi:10.3389/fphar.2020.00344)
Supplement: Supplementary file 6 [file Table_3.pdf]

**Supplementary Table 3.**

| **Antibody type** | **Clonality** | **Target** | **Host** | **Company** | **Reference #** | **Lot** | **Dilution** |
|---|---|---|---|---|---|---|---|
| Primary | Polyclonal | Akt | Rabbit | Cell Signaling | 9272 | 25 | 1:500 |
| Primary | Monoclonal (clone D9E) | Phospho($Ser^{473}$)-Akt | Rabbit | Cell Signaling | 4060 | 19 | 1:500 |
| Primary | Monoclonal (clone 54D2) | S6 | Mouse | Cell Signaling | 2317 | 4 | 1:250 |
| Primary | Monoclonal (clone D57.2.2E) | Phospho($Ser^{235/236}$)-S6 | Rabbit | Cell Signaling | 4858 | 11 | 1:250 |
| Primary | Monoclonal (clone 4G-1E) | GSK3α/β | Mouse | Merck Millipore | 05-412 | 2303952 | 1:500 |
| Primary | Polyclonal | Phospho($Ser^{21/9}$)-GSK3α/β | Rabbit | Cell Signaling | 9331 | 15 | 1:250 |
| Primary | Polyclonal | β-actin | Rabbit | Abcam | ab8227 | 133004 | 1:20,000 |
| Primary | Monoclonal (clone AC-15) | β-actin | Mouse | Sigma Aldrich | A1978 | 104K4787 | 1:200,000 |
| Secondary | Polyclonal | Alexa Fluor 680 anti-Rabbit IgG (H+L) | Goat | Thermo Fisher Sc. | A21076 | 47704A | 1:4,000 |
| Secondary | Polyclonal | Alexa Fluor 680 anti-Mouse IgG (H+L) | Goat | Thermo Fisher Sc. | A21057 | 44888A | 1:4,000 |
| Secondary | Polyclonal | IRDye 800 Anti-Rabbit IgG (H+L) | Donkey | Rockland Immunoc. | 611-732-127 | 22882 | 1:5,000 - 1:20,000 |
| Secondary | Polyclonal | IRDye 800 Anti-Mouse IgG (H+L) | Donkey | Rockland Immunoc. | 610-731-124 | 18249 | 1:5,000 - 1:10,000 |

**Details of the antibodies used and their dilutions for human PFC experiments**

| **Antibody type** | **Clonality** | **Target** | **Host** | **Company** | **Reference #** | **Lot** | **Dilution** |
|---|---|---|---|---|---|---|---|
| Primary | Polyclonal | Akt | Rabbit | Cell Signaling | 9272 | 25 | 1:500 |
| Primary | Monoclonal (clone D9E) | Phospho($Ser^{473}$)-Akt | Rabbit | Cell Signaling | 4060 | 19 | 1:500 |
| Primary | Monoclonal (clone 54D2) | S6 | Mouse | Cell Signaling | 2317 | 4 | 1:500 |
| Primary | Monoclonal (clone D57.2.2E) | Phospho($Ser^{235/236}$)-S6 | Rabbit | Cell Signaling | 4858 | 11 | 1:500 |
| Primary | Monoclonal (clone 4G-1E) | GSK3α/β | Mouse | Merck Millipore | 05-412 | 2303952 | 1:500 |
| Primary | Polyclonal | Phospho($Ser^{21/9}$)-GSK3α/β | Rabbit | Cell Signaling | 9331 | 15 | 1:500 |
| Primary | Polyclonal | β-actin | Rabbit | Abcam | ab8227 | 133004 | 1:20,000 |
| Primary | Monoclonal (clone AC-15) | β-actin | Mouse | Sigma Aldrich | A1978 | 104K4787 | 1:100,000 |
| Secondary | Polyclonal | Alexa Fluor 680 anti-Rabbit IgG (H+L) | Goat | Thermo Fisher Sc. | A21076 | 47704A | 1:4,000 - 1:5,000 |
| Secondary | Polyclonal | Alexa Fluor 680 anti-Mouse IgG (H+L) | Goat | Thermo Fisher Sc. | A21057 | 44888A | 1:4,000 - 1:5,000 |
| Secondary | Polyclonal | Dylight 800 Anti-Rabbit IgG (H+L) | Donkey | Rockland Immunoc. | 611-745-127 | 22882 | 1:5,000 - 1:10,000 |
| Secondary | Polyclonal | Dylight 800 Anti-Mouse IgG (H+L) | Donkey | Rockland Immunoc. | 610-745-002 | 23294 | 1:5,000 - 1:10,000 |

**Details of the antibodies used and their dilutions for rat cortex experiments**

IgG = Immunoglobulin G; H+L = heavy and low chains; Immunoc. = Immunochemicals; Sc. = Scientific.
